# Worksite Characteristics and Environmental and Policy Supports for Cardiovascular Disease Prevention in New York State

**Published:** 2008-03-15

**Authors:** Ian Brissette, Brian Fisher, Deborah A Spicer, Lori King

**Affiliations:** Bureau of Chronic Disease Epidemiology and Surveillance, New York State Department of Health; School of Public Health, State University of New York at Albany, Albany, New York; Bureau of Health Risk Reduction, New York State Department of Health, Albany, New York; Bureau of Chronic Disease Epidemiology and Surveillance, New York State Department of Health, Albany, New York

## Abstract

**Introduction:**

Worksite policy and environmental supports that promote physical activity, healthy eating, stress management, and preventive health screenings can contribute to the prevention of cardiovascular disease and lower employer costs. This study examines the availability of these four categories of supports in a statewide survey of New York State worksites.

**Methods:**

In 2002, we recruited a statewide sample of worksites in New York State with 75 or more employees to participate in a mailed survey assessing worksite policy and environmental supports for wellness and health promotion. The overall response rate was 34.8%. The analysis included data from 832 worksites.

**Results:**

Worksite size was an independent predictor of health promotion supports with small (75–99 employees) and medium-small (100–199 employees) worksites reporting significantly fewer policy and environmental supports in all four categories than worksites with 300 or more employees. Worksites in which most employees were nonwhite reported fewer supports for physical activity, healthy eating, and stress management than worksites in which most employees were white. A wellness committee or wellness coordinator was associated with more health promotion supports, regardless of the size of the worksite or composition of its workforce.

**Conclusion:**

Worksites with fewer than 200 employees have an increased need for assistance in establishing environmental and policy supports promoting cardiovascular health. Worksites that have a wellness committee or coordinator are better able to establish and sustain supports with the potential to improve the health of their workers.

## Introduction

Worksite health promotion programs have the potential to reach large segments of the adult population and allow control over interpersonal, environmental, and organizational factors that influence health behavior (1). They are an important venue for cardiovascular disease prevention. Recent studies demonstrating that comprehensive worksite wellness programs can promote worker productivity and reduce costs have increased employers' interest in implementing such programs (2-5). This interest has generated opportunities for the public health sector to partner with employers in the design and delivery of worksite health promotion activities. Moreover, the interest has increased the need for information about existing on-site wellness supports and identification of where the need for intervention is greatest and where intervention is likely to have the greatest impact. For this study, we used data from a population-based, statewide survey of worksites in New York State to characterize existing worksite supports for physical activity, healthy eating, stress management, and preventive health screenings and to identify the characteristics of worksites possessing these different types of wellness supports.

Our primary goal was to examine how worksite programs promoting on-site preventive health screenings relate to efforts focused on policy and environmental changes that support physical activity, healthy eating, and stress management. Efforts to prevent cardiovascular disease through worksite health promotion have traditionally focused on establishing environmental and policy changes that promote physical activity, healthy eating, and tobacco control (6,7). However, secondary prevention activities aimed at promoting blood pressure control and lowering cholesterol through on-site screening, referral, and treatment programs have increasingly become incorporated into worksite health promotion programs (8-10). Understanding the association between worksite preventive health screenings and efforts of employers to implement policy and environmental supports for physical activity and healthy eating could provide insight about how worksites are implementing primary and secondary prevention efforts. For example, if worksites implementing on-site screening programs also have greater policy and environmental supports for the primary prevention of cardiovascular disease, these employers may be implementing on-site screening programs as part of a comprehensive program.

A second goal was to examine the worksite and workforce characteristics associated with the availability of health promotion supports. Both national surveys and regional, state-based worksite surveys indicate that a worksite's size (i.e., number of employees), its administrative support for employee wellness, and its industrial classification are associated with the availability of on-site health promotion activities (11-15). Moreover, studies of the impact of worksite health promotion programs suggest that certain characteristics of the workforce — including race/ethnicity, degree of unionization, percentage of blue-collar workers, and sex (i.e., percentage male or female) — have implications both for the need for worksite health promotion activities and for how activities are delivered (15-20). In this study, we evaluated which of these worksite and workforce characteristics were independent predictors of worksite supports for healthy eating, physical activity, stress management, and preventive health screenings. Identifying which characteristics are associated with these categories of worksite health promotion activities would help to encourage the development of interventions targeted toward the worksites with the greatest need and tailored to their needs.

We used data from a statewide survey of New York State worksites. Since 1995, the New York State Department of Health (NYSDOH) has supported worksite health promotion programs emphasizing policies and environmental changes consistent with the social ecological model (1). The survey was intended to provide representative statewide data that could assist in identifying program needs and tracking the success of statewide efforts to support cardiovascular health in worksites.

## Methods

### Questionnaire development

We developed a questionnaire assessing worksite supports for cardiovascular health on the basis of the Heart Check, a validated 226-item inventory assessing worksite features relevant to cardiovascular health (21). We evaluated items for appropriateness using the question appraisal system (22), and members of the NYSDOH Healthy Heart Program reviewed items for content. In June 2001, 19 Healthy Heart Program worksite contractors administered the worksite wellness questionnaire at worksites where they had previously completed the Heart Check. The mean percentage agreement for items included in both instruments was 71% (range, 33%–100%). Items for which agreement was low were modified to better convey the worksite supports being assessed. The final questionnaire consisted of 21 main survey questions and 9 conditional questions on worksite supports in six areas: 1) general wellness and health promotion, 2) healthy eating, 3) physical activity, 4) tobacco use, 5) preventive health screening, and 6) stress management. Worksite representatives were asked to respond to the main survey questions using one of three responses (yes, no, "don't know") and estimate the percentage of workers who were women, blue collar, union members, white, African American, Native American, or Asian using one of the following seven categories: 0%, 1% to 10%, 11% to 25%, 26% to 50%, 51% to 75%, 76% to 100%, or "don't know."

### Sampling and survey administration

We acquired a database of companies with more than 75 employees from Dun and Bradstreet in August 2001, and it served as the sampling frame. We drew a stratified random sample to ensure representation from both private- and public-sector worksites, using random replacement for nonresponding and refusing worksites within private and public sectors until sufficient sample sizes were obtained. We administered the survey by mail using methods adapted from Dillman (23). From February 2002 through March 2003, we mailed surveys to 1833 private-sector and 794 public-sector companies. We included surveys received before April 2003 in the analysis. The overall response rate was 34.8%, with 33.6% of private-sector and 37.0% of public-sector worksites participating.

### Statistical analyses

First, we evaluated the characteristics of the worksites surveyed and prepared descriptive statistics. We used a chi-square test to determine on the basis of size which worksites were more likely to have missing data.

We then identified 21 discrete environmental and policy supports that would serve as count variables representing four categories of support: physical activity, healthy eating, stress management, and preventive health screenings. We calculated weighted mean estimates of the number of supports available at the worksites, and we determined simple correlations among the four categories of supports using SAS (SAS Institute, Inc, Cary, North Carolina). We used Pearson correlation coefficients to convey the association between categories.

Next, we used SUDAAN Release 7.5 (Research Triangle Institute, Research Triangle Park, North Carolina) to calculate the weighted estimates of the mean number of various categories of wellness supports by worksite characteristic. These estimates combined information about the sampling design and sample weights to generate accurate estimates of the means and 95% confidence intervals. The sample weights included information about the probability of selection, a nonresponse adjustment, and a poststratification adjustment.

Finally, we used SUDAAN to construct linear models examining which worksite characteristics were independent predictors of categories of worksite supports for health promotion and cardiovascular health. The linear models used generalized estimating equations and calculated estimates of the regression coefficients using the Binder method (24,25).

## Results

Table 1 lists the characteristics of the worksites participating in the survey. Information on geographic region, industry, and number of employees was extracted from the Dun and Bradstreet database. Information about race, sex, percentage of blue-collar workers, percentage of full-time employees, and degree of unionization was obtained from the mailed questionnaire. Although not all worksite participants answered all questions on workforce demographics, 504 worksites (61%) answered all such questions. Worksites with 75 to 99 employees and those with 300 or more employees were significantly more likely to have missing data than worksites with 100 to 199 or 200 to 299 employees (Χ^2^_3_ = 7.7; *P* = .05).

Table 2 presents the items used to create the four variables (physical activity, healthy eating, stress management, and preventive health screenings), weighted mean estimates of the number of supports available at the worksites, and the simple correlations among the four categories of supports. Of the 21 discrete environmental and policy supports reflected in the four count variables, the average worksite in the sample reported only 5.64 supports. The worksites reported having a greater number of nutrition (1.82) and stress management (1.93) supports than physical activity supports (0.96). On average, worksites reported holding fewer than one preventive health screening (0.93) in the previous year, and only 36% of the worksites in the sample reported holding one or more screenings. The two types of preventive screenings worksites held most frequently were blood pressure screenings (reported to be held at least once during the previous year by 23.8% of worksites) and general health risk appraisals (reported to be held at least once during the previous year by 17.1% of worksites). As indicated in Table 2, the four categories of wellness supports were all positively and significantly correlated. The strength of the correlation between the number of health screenings held at the worksite and the other three categories of wellness supports did not differ in magnitude from any of the correlations among the three other categories of wellness supports.

Table 3 presents weighted estimates of the mean number of wellness supports for healthy eating, physical activity, stress management, and preventive health screenings by worksite and workforce characteristics. Table 4 presents the results of multivariate regression models evaluating which of these characteristics were independent predictors of the four categories of supports for cardiovascular disease prevention. Both tables include information from worksites with missing data on one or more of the worksite or workforce characteristics. Including this information enabled us to retain worksites in our descriptive tables and statistical models and provided an appropriate and conservative test of whether the other variables in the model were significant predictors of the outcome variable. We also performed analyses that eliminated worksites with missing data, producing results that did not differ from the results reported in this paper (analyses available upon request).

### Worksite characteristics

Worksite size and the presence of worksite administrative supports for wellness were associated with the availability of health promotion supports in the descriptive analyses and multivariate models. Small worksites (75–99 employees) reported significantly fewer healthy eating supports than medium-small (100–199 employees), medium-large (200–299 employees), and large (≥300 employees) worksites. Small worksites also reported fewer stress management supports than medium-large or large worksites and fewer physical activity supports and preventive health screenings than large worksites. Medium-small worksites resembled small worksites in having fewer supports for healthy eating, physical activity, and stress management than large worksites. Worksite size remained a significant and independent predictor of each of the four categories of supports in our multivariate models, with small and medium-small worksites reporting significantly fewer supports for all four categories than large worksites.

Worksites with a wellness committee or wellness coordinator reported a greater number of all four categories of worksite health promotion supports than those without either of these administrative supports. Moreover, worksites having both types of administrative supports reported more supports for healthy eating and physical activity and more preventive health screenings than those having only a committee or a coordinator. The presence of one or more administrative supports for employee wellness was also associated with a greater number of all four categories of health promotion supports in our multivariate models, suggesting that the relationship could not be attributed to larger companies being more likely to have a wellness coordinator or committee.

### Workforce characteristics

The racial and sex composition of the workforce and the extent to which the workforce was unionized were significantly associated with the number of health promotion supports available at a worksite in both the descriptive analyses and multivariate models. Worksites in which most workers were nonwhite had fewer supports for healthy eating, physical activity, and stress management. This association remained significant in our multivariate models. Worksites in which most workers were women had more environmental policy supports for physical activity, healthy eating, and stress management in our descriptive analyses, but only the association with healthy eating supports remained significant in the multivariate models. Both the descriptive and multivariate models demonstrated that worksites in which most of the workforce was unionized had more stress management supports. However, the multivariate models indicated worksites with a greater percentage of union members held fewer preventive health screenings during the previous year. The blue-collar status of the workforce was associated with the availability of physical activity supports in our descriptive analyses but was not associated with the availability of any of the categories of health promotion supports in the multivariate models.

## Discussion

The findings from our statewide survey reinforce the need for additional efforts to promote worksite health promotion among New York State worksites. One strength of the study is that the findings are based on a statewide, population-based sample of New York State worksites. From 1985 through 1999, three national surveys of worksite health promotion activities and supports took place (14,15,26). These surveys continue to provide useful data for states planning worksite health promotion efforts. However, because of regional differences in industry and workforce populations, they cannot provide a substitute for statewide data in the planning of statewide and regional initiatives. The NYSDOH has routinely collected data on the health promotion activities of worksites targeted by its Healthy Heart Worksite Wellness Program for the purpose of evaluating program efforts (21). However, our study represents the first attempt to collect statewide data using a population-based survey.

Our primary goal was to examine how worksite programs promoting on-site preventive health screenings relate to efforts concentrating on policy and environmental changes that support physical activity, healthy eating, and stress management. Worksites holding on-site preventive health screenings during the previous year tended to have more policy and environmental supports for physical activity, healthy eating, and stress management. Moreover, the strength of the correlations among the four categories of supports was equivalent, implying preventive health screenings are incorporated into worksite health promotion programs much as traditional efforts to lower stress, increase physical activity, and encourage healthy eating are incorporated. This finding is consistent with a recent review concluding that certain company attributes — such as leadership support — provide an infrastructure that supports all types of worksite health promotion initiatives (5).

A second goal was to examine the worksite and workforce characteristics associated with the availability of health promotion supports. We replicated the results of past studies demonstrating that smaller worksites have the fewest worksite health promotion supports (11-13). Although the discrepancy in the availability of wellness supports was greatest between small worksites and large worksites, medium-small companies also had significantly fewer health promotion supports than the larger worksites. This difference suggests that the public health sector should consider developing programs that assist medium-small worksites in their efforts to implement worksite health promotion. Targeting medium-small worksites instead of small worksites would allow programs to reach more people. Another benefit of targeting medium-small worksites is that they may be more likely than small worksites to have the infrastructure required to sustain policy and environmental changes.

Our analyses demonstrated that worksites with a wellness committee or wellness coordinator had more supports in all four support categories, regardless of the size or composition of the workforce. Furthermore, worksites with both a committee and coordinator had more supports than those with only one. The presence of two administrative supports may be robust predictors of supports because they encompass both employer and worker support for health promotion, both of which have been demonstrated to be critical to the success of worksite health promotion programs (5,27). Establishing a wellness coordinator or committee would benefit worksites of all sizes in their efforts to implement and sustain health promotion efforts.

The racial and sex composition of the workforce and the extent to which a workforce was unionized were also associated with the type and extent of the health promotion supports. Our finding that worksites in which the workforce was mostly nonwhite had fewer healthy eating, physical activity, and stress management supports contributes to the body of literature indicating significant disparities in health and in the availability of health-related supports by race and ethnicity (18,28). Our finding also reinforces the suggestion that health disparities in the population are rooted, in part, in the environments in which different segments of the population live and work (29). Our finding that worksites in which the workforce was mostly women had more supports for healthy eating is consistent with past studies demonstrating an association between a high proportion of female employees, stringent worksite smoking policies, and worksite stress management supports (16,19,20). In the absence of information about a worksite's health promotion supports, health promotion program planners could use information on the percentage of nonwhite workers, and to a lesser extent, female workers as proxy information for determining need.

We found that greater union representation was associated with more stress management supports but fewer on-site preventive screenings in the past 12 months. A previous survey of New York State worksites, however, showed that greater union representation was associated with more supports for physical activity, screenings, and overall health promotion (13). One explanation for these opposing results is that whereas the previous survey was based on a convenience sample of worksites, the current survey was completed on a population-based, stratified random sample of worksites. Public health has an opportunity to mobilize state and local unions to play a more significant role in worksite health promotion. We can provide evidence on how worksite health promotion can improve the health of workers in a cost-effective way and equip them with other supports so that union leadership can communicate with the companies and organizations employing their members.

One limitation of our survey instrument was that its format led to missing data on workforce demographic characteristics. Although the missing data were not missing at random, the high rate of missing data on some items did not provide an alternative explanation for the findings reported. A second limitation is that the survey instrument included few questions related to the secondary prevention of cardiovascular disease and no questions related to stroke prevention. To address this limitation, the NYSDOH is repeating the statewide survey with a modified instrument that should provide a more comprehensive assessment. The follow-up survey will enable the NYSDOH Healthy Heart Program to determine changes in worksite health promotion supports that have occurred since this survey and will provide baseline data for tracking future worksite health promotion efforts related to blood pressure and cholesterol control and stroke prevention.

## Figures and Tables

**Table 1:** Characteristics of Worksites (N = 832) Participating in Survey of New York State Worksites, 2002^a^

Characteristic	No. (%) Worksites
**Region**	
New York City	165 (20.0)
New York State (not including New York City)	667 (80.0)
**Industry**	
Manufacturing	112 (13.5)
Transportation, communication, utilities	30 (3.6)
Retail trade	48 (5.8)
Finance, insurance, real estate	30 (3.6)
Services	295 (35.5)
Public administration	280 (33.7)
Other	37 (4.4)
**No. of employees**	
75-99	223 (26.8)
100-199	322 (38.7)
200-299	109 (13.1)
≥300	178 (21.4)
**% White employees**	
0-25	75 (11.0)
26-50	72 (10.6)
51-75	127 (18.7)
76-100	405 (59.6)
**% Female employees**	
0-25	171 (24.6)
26-50	159 (22.9)
51-75	229 (33.0)
76-100	135 (19.5)
**% Full-time employees**	
0-25	36 (5.0)
26-50	44 (6.2)
51-75	113 (15.8)
76-100	521 (73.0)
**% Blue-collar employees**	
0-25	302 (51.5)
26-50	64 (10.9)
51-75	122 (20.8)
76-100	98 (16.7)
**% Union-member employees**	
0-25	309 (44.0)
26-50	30 (4.3)
51-75	87 (12.4)
76-100	276 (39.3)

a Category values may not add to 832 because some worksites had missing data. Percentages may not total to 100.0% because of rounding.

**Table 2:** Support Items and Association^a^ Between Types of Support for Cardiovascular Disease Prevention Among Worksites (N = 832) Participating in Survey of New York State Worksites, 2002

Type of Support	Weighted MeanEstimates (95% CI)	Physical Activity	Healthy Eating	Stress Management	Health Screenings
Physical activity	0.96 (0.88-1.04)	—	0.39	0.33	0.28
Healthy eating	1.82 (1.70-1.94)	—	—	0.35	0.28
Stress management	1.93 (1.79-2.07)	—	—	—	0.29
Health screenings	0.93 (0.79-1.07)	—	—	—	—
**Support Items**					
**Physical activity**					
Written policy supporting exercise or physical activity during work time					
Exercise facility available or discounted or subsidized membership					
On-site physical activity-oriented program offered during the past 12 months					
Safe place for recreational walking at the worksite					
**Healthy eating**					
Three or more healthy eating options available at worksite					
Labels to identify healthier food choices					
Policy to make healthy food options available to employees					
On-site programs on nutrition or weight management during the past 12 months					
**Stress management**					
Employee assistance program					
Formal employee grievance procedure					
Management training on stress-related issues					
Organized social events for employees					
Break room or lounge other than cafeteria or lunchroom					
**Health screenings**					
Health risk appraisal					
Blood pressure					
Cholesterol					
Physical fitness tests					
Body fat or body weight screening					
Periodic health or physical examination					
Diet or nutrition evaluation					
Blood glucose measurement					

CI indicates confidence interval.

a Pearson correlation coefficients were used to convey the association between categories of supports.

**Table 3:** Estimated Number of Supports (Weighted Means) for Cardiovascular Disease Prevention Among Worksites (N = 832) Participating in Survey of New York State Worksites, 2002, by Worksite and Workforce Characteristics

Characteristic	Healthy EatingMean (95% CI)	Physical ActivityMean (95% CI)	Stress ManagementMean (95% CI)	Preventive Health ScreeningsMean (95% CI)
**Industry type**				
Manufacturing	2.03 (1.83-2.23)	1.00 (0.84-1.16)	1.98 (1.70-2.26)	0.84 (0.46-1.22)
Transportation, communications, utilities	1.47 (0.97-1.97)	0.80 (0.42-1.18)	2.26 (1.60-2.92)	0.90 (0.46-1.34)
Retail trade	1.74 (1.32-2.16)	0.55 (0.31-0.79)	1.55 (1.05-2.05)	0.55 (0.15-0.95)
Finance, insurance, real estate	1.70 (1.18-2.22)	0.97 (0.61-1.33)	1.44 (0.86-2.02)	1.00 (0.28-1.72)
Services	1.96 (1.80-2.12)	1.06 (0.92-1.20)	2.10 (1.90-2.30)	1.06 (0.84-1.28)
Public administration	1.55 (1.39-1.71)	1.27 (1.13-1.41)	2.33 (2.13-2.53)	1.05 (0.85-1.25)
Other	1.21 (0.79-1.63)	0.75 (0.39-1.11)	1.23 (0.69-1.77)	0.70 (0.12-1.28)
**No. of employees**				
75-99	1.37 (1.17-1.57)	0.83 (0.67-0.99)	1.68 (1.38-1.98)	0.68 (0.30-1.06)
100-199	1.76 (1.62-1.90)	0.90 (0.78-1.02)	1.75 (1.55-1.95)	0.73 (0.29-1.17)
200-299	2.23 (1.95-2.51)	0.94 (0.74-1.14)	2.27 (1.91-2.63)	1.08 (0.68-1.48)
≥300	2.57 (2.33-2.81)	1.35 (1.13-1.57)	2.56 (2.30-2.82)	1.69 (1.25-2.13)
**Administrative wellness support**				
None	1.50 (1.36-1.64)	0.72 (0.62-0.82)	1.56 (1.40-1.72)	0.44 (0.32-0.56)
Committee or coordinator	2.20 (1.96-2.44)	1.21 (1.03-1.39)	2.47 (2.15-2.79)	1.51 (1.11-1.91)
Committee and coordinator	2.66 (2.40-2.92)	1.73 (1.45-2.01)	2.87 (2.53-3.21)	2.29 (1.81-2.77)
**% Blue-collar employees**				
0-50	1.80 (1.64-1.96)	1.04 (0.90-1.18)	1.95 (1.73-2.17)	1.01 (0.77-1.25)
51-100	1.73 (1.49-1.97)	0.73 (0.57-0.89)	1.96 (1.66-2.26)	0.75 (0.51-0.99)
Missing data	1.93 (1.73-2.13)	1.06 (0.88-1.24)	1.88 (1.64-2.12)	0.96 (0.68-1.24)
**% White employees**				
0-50	1.52 (1.26-1.78)	0.72 (0.52-0.92)	1.74 (1.42-2.06)	0.88 (0.58-1.18)
51-100	1.93 (1.81-2.05)	1.07 (0.89-1.25)	2.09 (1.93-2.25)	0.87 (0.69-1.05)
Missing data	1.92 (1.62-2.22)	0.96 (0.68-1.24)	1.76 (1.36-2.16)	1.16 (0.68-1.64)
**% Union-member employees**				
0-50	1.76 (1.60-1.92)	0.95 (0.83-1.07)	1.74 (1.54-1.94)	0.95 (0.83-1.07)
51-100	1.91 (1.71-2.11)	0.93 (0.79-1.07)	2.26 (2.02-2.50)	0.92 (0.78-1.06)
Missing data	1.77 (1.47-2.07)	1.04 (0.80-1.28)	1.83 (1.41-2.25)	1.20 (0.78-1.62)
**% Female employees**				
0-50	1.54 (1.34-1.74)	0.80 (0.66-0.94)	1.74 (1.50-1.98)	0.81 (0.55-1.07)
51-100	1.97 (1.81-2.13)	1.01 (0.89-1.13)	2.06 (1.86-2.26)	0.91 (0.71-1.11)
Missing data	2.01 (1.69-2.33)	1.17 (0.95-1.39)	2.01 (1.79-2.23)	1.29 (0.83-1.75)

CI indicates confidence interval.

a Weighted means differ significantly at *P* < .05 within a worksite characteristic category (except industry) for a given type of support.

**Table 4:** Summary of Multivariate Models Examining Worksite Characteristics Associated With Categories of Support for Cardiovascular Disease Prevention Among Worksites (N = 832) Participating in Survey of New York State Worksites, 2002

Characteristic	Healthy Eating Model R^2^ = 0.28		Physical Activity Model R^2^ = 0.22		Stress Management Model R^2^ = 0.20		Preventive Screenings Model R^2^ = 0.22	
	β	*t*	β	*t*	β	*t*	β	*t*
**Industry type**								
Manufacturing	0.58^a^	4.29^a^	−0.18	1.51	−0.15	0.83	−0.25	1.10
Transportation, communication, utilities	0.14	0.59	−0.33	1.88	0.13	0.44	−0.29	1.12
Retail trade	0.61^a^	3.36^a^	−0.48	3.33^a^	−0.23	0.96	−0.27	1.19
Financials	0.39	1.79	−0.27	1.61	−0.55	1.78	−0.02	0.08
Services	0.57^a^	4.56^a^	−0.11	1.06	−0.01	0.10	0.15	0.98
Other	0.03	0.19	−0.32^a^	2.01^a^	−0.69^a^	2.56^a^	−0.18	0.58
Public administration	Referent group							
**No. of employees**								
75-99	−0.98^a^	6.23^a^	−0.33^a^	2.53^a^	−0.57^a^	3.18^a^	−0.72^a^	3.33^a^
100-199	−0.66^a^	4.81^a^	−0.31^a^	2.59^a^	−0.57^a^	3.65^a^	−0.69^a^	3.23^a^
200-299	−0.27	1.43	−0.26	1.80	−0.17	0.82	−0.40	1.54
≥300	Referent group							
**Administrative wellness support**								
None	−1.00^a^	6.66^a^	−0.93^a^	6.16^a^	−1.16^a^	6.36^a^	−1.74^a^	7.23^a^
Committee or coordinator	−0.42^a^	2.54^a^	−0.47^a^	2.82^a^	−0.33	1.54	−0.71^a^	2.39^a^
Committee and coordinator	Referent group							
**% Blue-collar employees**								
Missing data	0.08	0.54	0.21	1.64	−0.10	0.47	−0.14	0.75
0-50	−0.10	0.81	0.16	1.61	−0.10	0.57	0.01	0.04
51-100	Referent group							
**% White employees**								
Missing data	−0.43^a^	1.98^a^	−0.63^a^	4.00^a^	−0.94^a^	3.53^a^	−0.26	0.72
0-50	−0.39^a^	3.17^a^	−0.26^a^	2.51^a^	−0.37^a^	2.29^a^	0.06	0.36
51-100	Referent group							
**% Union-member employees**								
Missing data	−0.27	1.43	0.01	0.06	−.30	1.21	0.39	1.74
0-50	−0.05	0.45	0.12	1.37	−0.30^a^	2.01^a^	0.38^a^	2.51^a^
51-100	Referent group							
**% Female employees**								
Missing data	0.34	1.44	0.54^a^	3.07^a^	0.72^a^	2.32^a^	0.48	1.35
0-50	−0.26^a^	2.27^a^	−0.06	−0.63	−0.17	1.03	0.10	0.56
51-100	Referent group							

a β coefficients and *t* tests indicate a coefficient is significantly different from the referent group at *P* < .05.
